# A Sustainable Approach to Valuable Polyphenol and Iridoid Antioxidants from Medicinal Plant By-Products

**DOI:** 10.3390/antiox13081014

**Published:** 2024-08-20

**Authors:** Filippo Marchetti, Irene Gugel, Stefania Costa, Anna Baldisserotto, Alberto Foletto, Ilenia Gugel, Erika Baldini, Stefano Manfredini, Silvia Vertuani

**Affiliations:** 1 Department of Life Sciences and Biotechnology, Section of Medicines and Health Products, University of Ferrara, Via Fossato di Mortara 17–19, 44121 Ferrara, Italy; filippo.marchetti@unife.it (F.M.); irene.gugel@unife.it (I.G.); anna.baldisserotto@unife.it (A.B.); ilenia.gugel@unife.it (I.G.); erika.baldini@unife.it (E.B.); silvia.vertuani@unife.it (S.V.); 2 Department of Chemical, Pharmaceutical and Agricultural Sciences, University of Ferrara, Via L. Borsari 46, 44121 Ferrara, Italy; 3 Pharmacy A. Foletto, Via Nuova 4, 38067 Ledro, Italy; info@foletto.net

**Keywords:** upcycling, by-product, medicinal plant, polyphenols, iridoids, ultrasound-assisted extraction, natural deep eutectic solvents, GAPI, AGREEprep, AGREE

## Abstract

Supply chain waste gives rise to significant challenges in terms of disposal, making upcycling a promising and sustainable alternative for the recovery of bioactive compounds from by-products. Lignocellulosic by-products like STF231, which are derived from the medicinal plant extract industry, offer valuable compounds such as polyphenols and iridoids that can be recovered through upcycling. In an unprecedented study, we explored and compared conventional hydroethanolic extraction, ultrasound hydroethanolic extraction, and natural deep eutectic solvents–ultrasound extraction methods on STF231 to obtain extracts with antioxidant activity. The extraction profile of total polyphenols (TPCs) was measured using the Folin–Ciocalteu test and the antioxidant capacity of the extracts was tested with FRAP and DPPH assays. HPLC-UV was employed to quantify the phenolic and iridoid markers in the extracts. Additionally, the sustainability profile of the process was assessed using the green analytical procedure index (GAPI), AGREEprep, and analytical GREEnness metric approach (AGREE) frameworks. Our findings indicate that a choline chloride and lactic acid mixture at a 1:5 ratio, under optimal extraction conditions, resulted in extracts with higher TPC and similar antioxidant activity compared with conventional hydroethanolic extracts. The innovative aspect of this study lies in the potential application of sustainable upcycling protocols to a previously unexamined matrix, resulting in extracts with potential health applications.

## 1. Introduction

Polyphenols and iridoids are important classes of plant secondary metabolites with significant antioxidant capacities and therapeutic potentials. Polyphenols are known for their extensive health benefits and interactions with gut microbiota, while iridoids are valued for their multitarget properties against a range of diseases and their role in plant defense systems [1,2]. 

The topic of waste generation from industries, industrial food production, agriculture, and biological processes has acquired significant attention due to the challenges associated with by-product disposal. These concerns have encouraged innovative research to shift towards a circular bioeconomy, aiming to address and reduce the problems related to waste residues through sustainable practices and resource efficiency [3].

Supply chains are often criticized for the large amounts of waste they produce, which can create significant disposal challenges. However, it is essential to recognize that raw materials for food and health products, even for small and medium-sized manufacturing facilities, generate by-products that can be rich in valuable substances that can be recovered and repurposed. One solution that can be employed on both a small and large scale to reduce the issue of supply chain by-product management and recover valuable compounds is upcycling. This process involves transforming waste into the starting point for new technological cycles that generate new materials and bio-based chemicals. By adopting this approach, it is possible to reduce the environmental impact of supply chains and create economic opportunities through the production of valuable goods [4,5].

According to the waste-to-value concept, by-products can acquire new life as they become a renewed resource of high added-value raw materials [6].

The exploitation of by-products within the agri-food sector can entail various processing techniques designed to isolate an array of plant metabolites. However, the primary emphasis is on dietary fibers, edible oils, essential oils, and classes of valuable chemicals, including potent antioxidant phenols and polyphenols [7], which can be recovered for different applications involving cosmetic, pharmaceutical, and food preparations thanks to their multiple beneficial effects [8,9]. Extraction techniques hold great significance in obtaining biologically relevant molecules by capitalizing on plant biomass. However, a standardized method for the recovery of target molecules from supply chain by-products has not yet been established. Therefore, it is essential to investigate suitable extraction techniques that are effective and selective towards the analytes of interest while minimizing environmental impact, in alignment with the principles outlined in the circular bioeconomy guidelines [6]. Generally, conventional solid–liquid extraction (CE) methods, such as batch extraction, maceration, and Soxhlet extraction, are the most widely used. CE are based on the use of organic solvents, which are often highly flammable and toxic, and their manufacture depends on fossil resources [8,10]. Various limitations are associated with conventional methods, such as extended extraction times, considerable usage of solvents with low ecological compatibility, and substantial energy demands [11,12]. In recent years, novel phytochemical extraction techniques have emerged as alternatives to conventional methods, including ultrasound-assisted extraction (UAE) [13]. Furthermore, the use of natural deep eutectic solvents (NADES) in extraction processes can significantly reduce the energy and time required compared with conventional methods, while also providing the advantage of utilizing environmentally friendly solvents that pose no toxicological risks to humans or the environment [14]. The application of UAE and NADES in the field of biomass valorization has been successful, as these techniques have demonstrated their ability to extract target molecules with the same efficiency as conventional methods [15,16].

Taking everything into account, the objective of this study was to upcycle the STF231 by-product, a residual vegetable product typically discarded, produced by a reputable local company that specializes in the production of a traditional bitter tincture made using ancient recipes and commercially available in Europe for over a century. The aim was to utilize the residues of expensive medicinal plants, which are still rich in polyphenols and bitter compounds with potent antioxidant activity, by comparing conventional hydroethanolic extraction methods with novel extraction techniques, such as hydroethanolic and NADES-mediated UAE. To the best of our knowledge, this approach was being attempted on such materials for the first time. Therefore, the main variables affecting extraction efficiency were investigated, and the extracts were subjected to quantitative analysis of polyphenols and analysis of antioxidant potential.

All the extracts were processed under HPLC analysis for quantification of seven phenolic compounds and four iridoid glycosides markers (swertiamarin, amarogentin, gentiopicroside, and sweroside), responsible for the characteristic bittering flavor of the officinal tincture. It is important to note that, during the last decade, bitter taste has been extensively studied, as it can be positively correlated with the treatment of pathological alterations of the body, such as regulation of food intake and obesity, body mass, onset of metabolic, dysbiotic, and cardiovascular disorders [17,18]. Focus has been placed on swertiamarin, gentiopicroside, amarogentin, and sweroside as they are mediators of interesting known biological activities, such as antipyretic, cholagogue, choleretic, and hepatoprotective effects, along with extraoral effects involving smooth muscle and skin [19]. The unique aspect of this project is the utilization of the STF231 by-product, which is derived from the pharmaceutical plant extract industry and has not been investigated previously. By employing environmentally friendly extraction techniques on this plant material, we conducted a thorough evaluation to assess analysis of the content and sustainability of the extraction process. This approach represents a pioneering effort in the field.

## 2. Materials and Methods

### 2.1. Materials

Plant material was dried in ARGO LAB TCF50 (Argolab, Italy). Stirring and heating plates (VELP Scientifica, Usmate Velate, Italy) were used to set up conventional hydroethanolic extractions. A heating bath with a Ceia CP104 (Ceia, Italy) ultrasound source was used to perform the ultrasound-mediated extractions. A NEYA 8 (REMI Sales & Engineering Ltd., Goregaon, Mumbai, India) centrifuge was used to centrifuge the samples. A UV–6300PC (Avantor VWR, Milano, Italy) spectrophotometer was used in the spectrophotometric determinations of the Folin–Ciocalteu, DPPH, and FRAP assays. Viscosity of NADES was measured with a Brookfield viscosimeter DV2T (AMETEK Brookfield, MA, USA. All reagents and standards were purchased from Sigma Aldrich (St. Louis, MO, USA).

### 2.2. Plant Material

Twenty-five kg from several batches of by-product STF231 was kindly provided by a local company (A. Foletto Pharmacy, Trento, Italy, Est. 1866) and, to facilitate the storage process and avoid environmental microbial contamination or degradation, the plant material was dried in a ventilated desiccator at 37 °C for 24 h. The matrix was then pulverized through a laboratory blade homogenizer and stored in jars in 50 g aliquots, protected from light sources at room temperature until treatment.

### 2.3. Preliminary Plant Material Analysis

The plant material was characterized in terms of total nitrogen and crude protein content [20], crude fats [21], total ashes [22], moisture [23], and total cellulose [24]. Sugar profile was performed according to Romani et al. [25]. The shape and surface morphological characteristics of the STF231 matrix were investigated using a Zeiss GeminiSEM 460 scanning electronic microscope under the conditions previously reported [26].

### 2.4. Extractions

#### 2.4.1. Conventional Hydroethanolic Extraction

Stock solutions were prepared with concentrations of 25%, 50%, 75%, and 96% *v*/*v* ethanol in water according to Nastasijević et al. [27]. The process of extraction involved combining 1:10 *w*/*v* plant material with a hydroethanolic mixture at various ethanol-to-water ratios. The extractions were carried out simultaneously for a period of 48 h at temperatures of 25 °C, 40 °C, 60 °C, and 70 °C under reflux conditions to prevent evaporation and maintain a constant solid-to-solvent ratio and extracting mixture composition. After the 48 h extraction period, each sample was allowed to cool to room temperature, and centrifugation at 6000 rpm for 10 min was performed to separate the matrix residues from the supernatant. The obtained extracts were stored at −18 °C in the dark until they were analyzed further.

#### 2.4.2. Hydroethanolic Ultrasound-Assisted Extraction

Each extraction was evaluated at various plant material/solvent ratios, including 1:10, 1:20, 1:30, 1:40, and 1:50, using 25%, 50%, 75%, and 96% *v*/*v* ethanol in water. The extraction time was assessed from 5 to 70 min, and all samples were processed at distinct operating temperatures ranging from 25 °C to 70 °C [13]. Extraction processes were performed in a thermostatic ultrasonic bath with a fixed frequency of 37 kHz, maintained constant throughout the procedure. All hydroethanolic ultrasound-assisted extractions (EtOH UAE) were carried out in triplicate by using 15 mL conical centrifuge tubes with an operating volume of 8 mL and avoiding exposure to light. Each extract underwent centrifugation at 6000 rpm for 10 min to separate the plant matrix and was subsequently stored at −18 °C for further analysis.

#### 2.4.3. Natural Deep Eutectic Solvent Coupled with Ultrasound-Assisted Extraction

Three different natural deep eutectic solvents were formulated and utilized as extracting agents for treating plant by-products, following the guidelines outlined by Dai et al. [28], with modifications (Table 1). The first natural deep eutectic solvent (NADES) tested was a combination of citric acid and choline chloride (ChCl:CA) at a 1:2 ratio, as suggested by Gómez-Urios et al. [29]. To prepare this solvent, the components were vigorously stirred and heated at 70 °C for an hour until a transparent brownish viscous liquid was obtained. Aliquots of water, at percentages of 20%, 30%, and 40% *w*/*v*, were then added to the mixture to reach the appropriate dilution. Additionally, a 1:2 mixture of sucrose and citric acid (Su:CA) [30] and lactic acid with choline chloride (ChCl:LA) at a 1:5 ratio were also tested. These solvents were prepared by heating and stirring under the same conditions mentioned above. Each NADES mixture was characterized using FTIR to confirm the formation of hydrogen bonds typical of the eutectic mixtures thus obtained.

#### 2.4.4. NADES Ultrasound-Assisted Extraction

Each eutectic mixture was evaluated at solid-to-solvent ratios of 1:10, 1:20, 1:30, 1:40, and 1:50, within a total working volume of 8 mL contained in conical centrifuge tubes. All extractions were performed in a thermostatic bath at temperatures of 25 °C, 40 °C, 50 °C, and 60 °C, each lasting for 60 min, with the application of ultrasound at a frequency of 37 kHz at least in triplicate. At the end of the extraction cycle, mixtures underwent centrifugation for 10 min at 6000 rpm, and the supernatant was separated from the solid plant fraction. The extracts were then stored at −18 °C in the dark until subsequent characterizations.

### 2.5. Determination of Total Polyphenol Content

The Folin–Ciocalteu method [31] was employed with slight modifications for the determination of total polyphenol content in the extracts. Briefly, a calibration curve was obtained by diluting a gallic acid standard solution within the range of 1–20 μg. Each extract and standard solution underwent treatment with a diluted aqueous Folin–Ciocalteu reagent, followed by incubation in the dark for 5 min, post-vortexing each test sample for 10 s. Subsequently, 300 μL of a 20% *w*/*v* Na_2_CO_3_ solution was added to each sample, and the mixture was incubated in the absence of light sources for 90 min. Finally, the absorbance at a wavelength of 765 nm was recorded against a blank sample consisting of distilled water. The results for each sample were expressed in micrograms of gallic acid equivalents per mL of extract (μg GAE/mL) as the average of at least one test triplicate, as reported in Baldisserotto et al. [32].

### 2.6. DPPH Antioxidant Activity

The DPPH assay [33] provides a rapid and straightforward means to assess the scavenging activity of a target compound by measuring its ability to reduce the stable DPPH radical in 1,1-diphenyl–2-picrylhydrazyl. The determination of antioxidant capacity relies on observing a color change in the test solution, transitioning from purple to light yellow following the reaction between the sample or standards and DPPH. To quantitatively determine the percentage of antioxidant activity of the extracts against the DPPH radical, a spectrophotometric determination was conducted at a wavelength of 517 nm. To accurate determine antioxidant activity, Equation (1) was used:

(1) $$\mathrm{IC}\;\%=1-\left\lfloor \frac{\left(A_{1}-A_{2}\right)}{A_{0}}\right\rfloor \times 100$$

where A_0_ represents the absorbance of control without sample, A_1_ is the absorbance of sample, and A_2_ is the absorbance without DPPH. The assay was conducted by preparing a standard solution of DPPH in methanol and adding 0.750 mL of this solution to 1.5 mL of extract. The mixture was vortexed and incubated for 30 min in the dark, after which the absorbance was recorded.

### 2.7. FRAP (Ferric-Reducing Antioxidant Power)

Antioxidant power was evaluated through FRAP [34], which quantifies the reducing capacity of a test compound on the Fe(III) ion [30] in Fe(II) in an acidic environment and in the presence of 2,4, 6-tripyridyl-s-triazine (TPTZ). The reduction of Fe(III)-TPTZ complex to the corresponding Fe(II)-TPTZ complex, exhibiting a distinct blue color with spectrophotometric absorption recorded at a maximum wavelength of 593 nm, occurs in the presence of antioxidants. To perform the FRAP test, a mixture is first prepared by combining 0.1 M pH 3.6 acetate buffer, 10 mmol/L TPTZ in 40 mmol/HCl, and 20 mmol/L ferric chloride at a 10:1:1 ratio. This solution is freshly prepared immediately before analysis. The execution of the FRAP test involves adding 1.9 mL of the aforementioned solution and 100 µL of the sample (or solvent for the blank sample). Subsequently, all samples were vortexed and incubated in dark for 10 min at 37 °C, and the absorbance was measured using a UV-Vis spectrophotometer. For quantification, a calibration curve was constructed using Trolox as a standard solution. The results were expressed as µmol of Trolox equivalent (TE) per g of the extract, providing a measure of the antioxidant capacity of the tested samples.

### 2.8. HPLC Polyphenol Profiling

Polyphenol profiling was obtained according to Baldisserotto et al. [32]. Briefly, it employed an Agilent 1100 Series HPLC System equipped with a G1315A DAD set at 254 ± 8 nm and separation was performed at room temperature on a Hydro RP18 Sinergi 80 Å column (4.6 × 250 mm, 4 µm) from Phenomenex (Torrance, CA, USA). The flow rate was set at 1.2 mL in^−1^ with a mobile phase consisting of 0.1% *v*/*v* TFA (A) in water and 0.1%*v*/*v* TFA in acetonitrile (B). Elution was performed in a gradient program as follows: 90%A–80%A in 5 min, isocratic condition with 80%A for 5 min, 80%A–20%A in 10 min, 20%A–90%A in 2 min. A standard solution and samples were previously filtered on a 0.22 μm CA syringe filter and injected in 5 μL volume for the analysis. A mix of standard compounds consisting of 0.312 mg mL^−1^ trans-ferulic acid, 1.58 mg mL^−1^ gallic acid, 0.23 mg mL^−1^ chlorogenic acid, 0.325 mg mL^−1^ rutin, 0.120 mg mL^−1^ caffeic acid, 0.5 mg mL^−1^ quercetin, and 0.086 mg mL^−1^ ellagic acid were prepared in 1:1 water/acetonitrile and properly diluted to obtain calibration curves.

### 2.9. HPLC Determination of Iridoids

HPLC analysis was performed by employing apparatus consisting of a Jasco HPLC system (Jasco, Easton, MD, USA) equipped with a Jasco RHPLC PU–4180 quaternary pump, a Jasco AS–4050 autosampler, a Jasco RI–4030 refractive index detector, and a Jasco UV–4070 UV/Vis detector. Separation was achieved by employing a stainless-steel C–18 reverse-phase column (150 × 4.6 mm) packed with 5 μm particles (Alltima HP C18 5 μm, Alltech Associates, Inc. Deerfield, IL, USA) and thermostated at 30 °C. A personal computer equipped with cromNAV version 2 software was used for chromatogram acquisition and data processing. Chromatographic conditions followed Aberham et al.’s method [35] with minor modifications. The elution process was performed using a gradient program, where the eluent mixture consisted of water acidified with 0.025% acetic acid (A) and a mixture of n-propanol/acetonitrile at a 1:1 ratio (B). The gradient program was organized as follows: 99%A–70%A for 20 min, 5%A for 1 min, maintaining isocratic conditions at 5%A for 4 min, 99%A for 1 min, and holding isocratic conditions at 99%A for 3.5 min. The flow rate was set at 1 mL/min, resulting in a total runtime of 30 min. The chromatogram was acquired at a wavelength of 232 nm. Prior to injection, each extract was filtered through a 0.22 µm cellulose acetate filter and injected at a volume of 5 µm. Quantification was performed by referring to peak area of standard swertiamarin, gentiopicroside, amarogentin, and sweroside solubilized in methanol and appropriately diluted to extrapolate calibration curves.

### 2.10. Green Assessment

In order to assess a sustainability profile of the proposed protocols, the green analytical procedure index (GAPI), AGREE, and AGREEprep tools were used [36,37,38]. GAPI and AGREEprep are two distinct tools used for evaluating the sustainability of various phases in the analytical process, including sample collection and determination. GAPI utilizes pentagrams and a color scale to indicate the sustainability of the process, while AGREEprep assigns a score ranging from 0 to 1 to evaluate the greenness of each phase. AGREE is similar to AGREEprep, but it focuses on the overall process and its adherence to the 12 principles of green analytical chemistry. These tools are widely used in the field and provide immediate, simple, and effective visual analysis of the critical points and elements of strength relating to the analyzed method.

## 3. Results and Discussion

### 3.1. Preliminary Plant Material Analysis

The STF 231 matrix displays a dark brown color and has a characteristic woody aromatic odor. As reported by the supplier, the qualitative composition of the botanical mixture includes *Alchemilla* L., *Citrus sinensis*, *Geum urbanum*, *Gentiana acaulis*, *Gentiana asclepiadea*, *Gentiana lutea*, and *Rheum* L. To evaluate its composition and determine whether there were significant amounts of valuable compounds within the by-product, a preliminary analysis was conducted, and the results are presented in Table 2.

**Figure 1:**
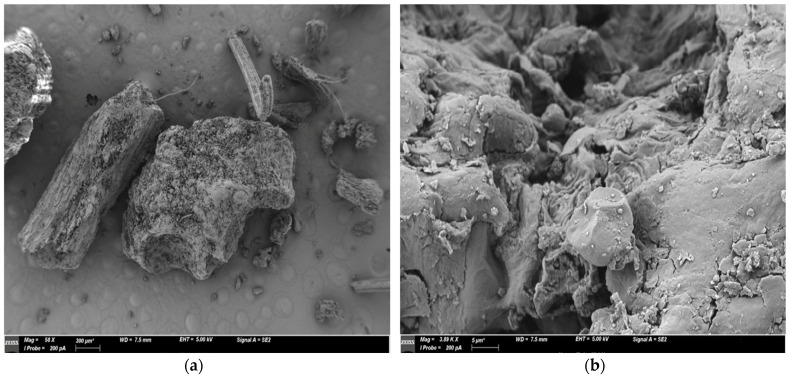
Microscopy of STF 231 particulate (**a**) and surface morphological analysis (**b**).

The vegetal matrix fragments depicted in Figure 1 exhibit a heterogeneous morphology, with STF231 featuring fibrous fragments and clear edges, likely resulting from the mechanical crushing process. The surface of the vegetal matrix particles is uneven and not smooth, displaying evident signs of fracture likely associated with the previous procedure for obtaining the bitter tincture. This morphological aspect is advantageous for the objectives of this study, as it provides a larger exposed surface that facilitates extraction mechanisms.

### 3.2. Total Polyphenol Content

#### 3.2.1. Conventional Hydroethanolic Extraction

Significant concentrations of polyphenols resulted in hydroethanolic extracts, performing extractions at 70 °C and ethanol concentrations equal to 50% *v*/*v*, allowing to obtain 7980.07 ± 55.02 μg GAE/mL, which resulted in the best extraction condition.

In this study, ethanol was examined as an extracting solvent due to its designation as an eco-friendly option and its suitability for use in food and cosmetic applications, while exhibiting a comparable performance to methanol [11].

Ethanol is a cost-effective, non-toxic, and reusable solvent that has the potential to serve as an environmentally friendly alternative for the extraction of bioactive compounds from solid by-products within a sustainable horizon. It is essential to evaluate the concentration of ethanol in extraction mixtures because changes in its composition can significantly impact the solubility of phenolic compounds and alter the properties of the extraction mixture [39].

**Figure 2:**
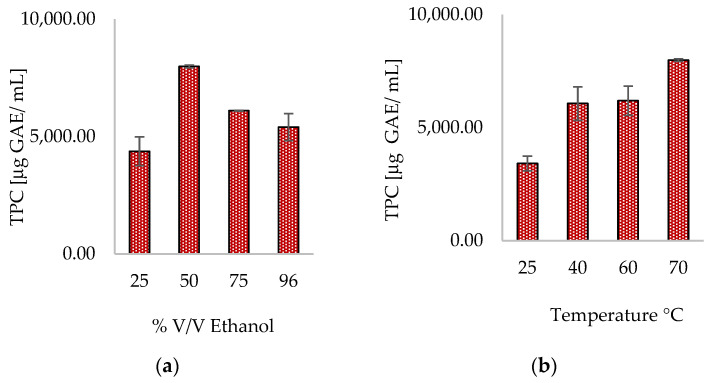
Effect of % ethanol in water (**a**) and temperature (**b**) on conventional hydroethanolic extraction.

The data presented in Figure 2a demonstrate a notable rise in TPC when the ethanol concentration was raised from 25% *v*/*v* to 50% *v*/*v*. However, as the concentration continued to increase to 75% and 96% hydroethanolic mixtures, there was a subsequent decline in TPC. Fifty percent hydroethanolic mixtures are known to have a biphasic effect [40] that can maximize TPC under selected conditions. The findings presented in Elboughdiri’s study are consistent with the observed trend [39]. The maximum concentration of polyphenols extracted from grape seed meal was obtained using a 50% *v*/*v* hydroethanolic mixture. However, lower and higher concentrations of ethanol in water resulted in a decrease in the recovery of polyphenols. The effect of temperature on the TPC profile is illustrated in Figure 2b. As the extraction temperature increased from 25 °C to 70 °C, the concentration of polyphenols in STF231 extracts also increased, with the maximum TPC obtained at 70 °C. The increase in extraction temperature can enhance the permeability of plant cell walls, allowing for greater extraction of phenolic compounds and facilitating mass transfer through the matrix. Additionally, the presence of 50% *v*/*v* ethanol in the extractant mixture amplifies this phenomenon by altering the structure of the cell membrane, increasing its permeability [41,42].

Similar findings in the literature [43,44] demonstrate that the extraction of polyphenols from citrus peel increases with rising temperature, reaching its peak at 80 °C.

#### 3.2.2. Hydroethanolic Ultrasound-Assisted Extraction

Ultrasound-assisted extraction involves the combination of physical mechanisms that promote cell disruption induced by cavitation. This approach leads to increased mass transfer of compounds from the cells to the solvent, thereby improving the efficiency of extraction [11].

**Figure 3:**
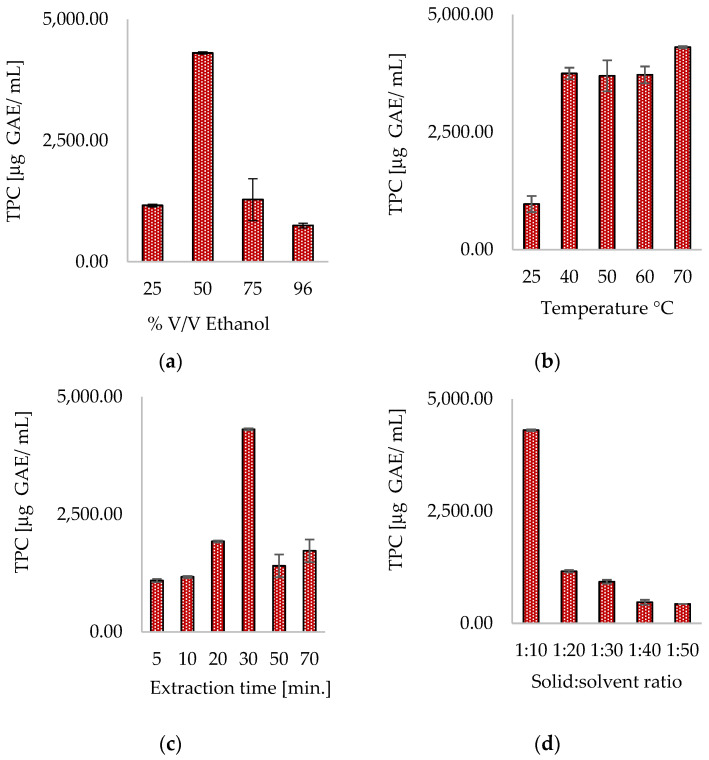
Effect on TPC of % ethanol (**a**), temperature (**b**), extraction time (**c**), and solid-to-solvent ratio (**d**) in ultrasound-assisted extraction at 37 kHz.

Upon assessment of variables in hydroethanolic UAE, it was observed that the highest yield of total phenolic content was achieved using 50% *v*/*v* ethanol in water, at a temperature of 70 °C for 30 min, and a solid-to-solvent ratio of 1:10 at a frequency of 37 kHz, resulting in a TPC of 4308.10 ± 20.63 μg GAE/mL. As demonstrated in Figure 3a, consistent with the results of conventional extraction, employing a 50% hydroethanolic mixture as the extracting solvent led to the highest extraction of polyphenols. Analysis of the ethanol concentration in the extraction solvent showed that using 50% ethanol consistently yielded the highest concentrations of polyphenols in the hydroethanolic ultrasound extracts. The influence of incubation time and extraction temperature were carefully examined in UAE. As depicted in Figure 3b, the TPC was relatively low at 25 °C. As the temperature increased from 25 °C to 40 °C, the amount of extracted polyphenol content also increased, remaining relatively steady until it reached 60 °C, and then showing a slight increase at 70 °C. The rise in temperature within the studied range led to an increase in the extraction of polyphenols, which could be attributed to the reduction in viscosity and density of the extracting mixture, allowing for deeper penetration into cells and enhancing mass transfer processes, and an increase in the solubility of polyphenols in the extraction mixture. Moreover, the ultrasound source’s cavitation effect intensifies this phenomenon, which leads to the disruption of matrix cells and enhances the diffusion of polyphenols into the solvent, resulting in a higher TPC [45,46,47].

The duration of incubation examined over a range of 5 to 70 min revealed a pattern, as depicted in Figure 3c. As the incubation time progressed, the level of extracted polyphenols demonstrated a notable increase, achieving its maximum after 30 min of incubation. However, extending the extraction process beyond 30 min led to a decline in the TPC. This can be attributed to the susceptibility of polyphenols to degradation when simultaneously subjected to thermal stress and cavitation effects. Prolonged extractions and relatively high temperatures can accelerate the breakdown of polyphenols, resulting in a reduction in polyphenol concentration after 30 min of extraction. Moreover, some researchers hypothesize that the decrease in TPC in UAE may be attributed to the sequestration of polyphenols by insoluble components, which are released from the matrix during prolonged extractions. This occurs after the disruption of cells, induced by cavitation effects [48]. The impact of the solid/solvent ratio is illustrated in Figure 3d. Notably, this research unveils a trend that contrasts with the typical findings in ultrasound extraction optimization studies, where an increase in the matrix-to-solvent ratio commonly leads to an increment in the concentration of extracted polyphenols. Thus, an increase in the ratio results in a gradient from the interior of the matrix to the solvent, which promotes enhanced mass transfer to the extraction environment and augments the quantity of extractable molecules. Furthermore, in UAE, the cavitation effect intensifies this phenomenon as the turbulent motions triggered by bubble collapse facilitate solvent contact with the material to be extracted [47]. Undoubtedly, the matrix-to-solvent ratio plays a crucial role in determining the best TPC of the resulting extract. As the ratio increases, the TPC tends to decrease. This observation can be rationalized by the prior extraction treatment that the matrix has already undergone to obtain the commercial bitter tincture. Thus, it is reasonable to assume that at, a ratio of 1:10, the matrix has reached the equilibrium necessary for effective polyphenol extraction. On the other hand, an increase in the solvent alone leads to the dilution of the extracted polyphenols, ultimately resulting in a decrease in the TPC.

#### 3.2.3. Natural Deep Eutectic Solvent Coupled with Ultrasound-Assisted Extraction

All eutectic mixtures underwent FTIR characterization prior to their use as extraction agents. As depicted in Figures S1–S3, a comparative analysis was conducted between the constituent components of each eutectic mixture used in this study and the mixture generated after formulation. In the FTIR spectra of each NADES, it was possible to observe broad peaks ranging from 3400 to 3600 cm^−1^, which corresponded with O-H stretching bonds, and between 1645 and 1658 cm^−1^, which were characteristic peaks that indicated the formation of hydrogen bonds between native components [49,50]. Various factors can influence the efficiency of NADES extraction, including the type of eutectic mixture, water content, and extraction temperature, which have been extensively examined [51].

**Figure 4:**
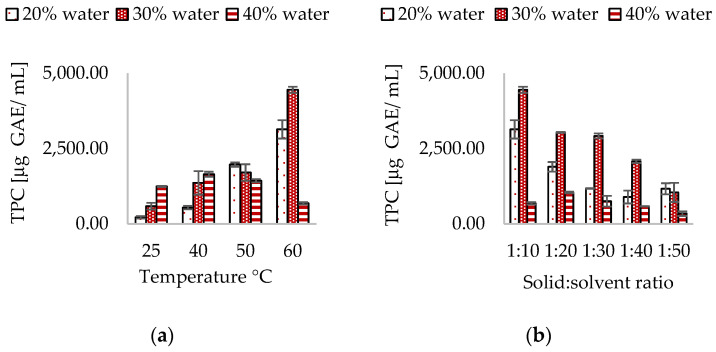
Effect of water content, (**a**) temperature, and (**b**) solid-to-solvent ratio in ChCl:CA NADES.

ChCl:CA allowed to achieve a TPC equal to 4454.02 ± 103.17 µg G.A.E/mL. The extraction process involved the presence of 30% *w*/*v* water, at a temperature of 60 °C, with a solvent ratio of 1:10, and 37 kHz sonication for 60 min, as depicted in Figure 4.

First of all, it is necessary to observe how the addition of water to the eutectic mixture resulted in a decrease in viscosity, which had significant practical implications that could alter the extraction profile of the analytes [52]. The water content in NADES allows them to be classified into ‘‘low water”, ‘‘medium water”, and ‘‘high water” [53,54,55]. Addition of water, even in small amounts up to 15% *w*/*w*, resulted in variations in the structure of the eutectic mixture. However, above 50% *w*/*w* of water, the hydrogen bonds formed between the hydrogen bond acceptor (HBA) and hydrogen bond donor (HBD) were gradually weakened; despite this, the supramolecular structures were preserved [28,54].

Additional water can ultimately lead to a decrease in the interactions between HBA and HBD, resulting in the formation of an aqueous solution wherein the NADES components are effectively dissolved [56].

In ChCL:CA ultrasound extracts, the optimal water content for maximum extraction of polyphenols was 30% *w*/*v*, which was observed to yield higher polyphenols. Conversely, TPC was lower at 20% and 40% *w*/*v* of water. At 20% *w*/*v* of water, high viscosity (η = 220 cPs) could interfere with the mass transfer process of phenols to the solvent, whereas at 40% *w*/*v*, the amount of water added significantly reduced the viscosity (η = 57.40 cPs), but also contributed to weakening the interactions that shaped the eutectic solvent’s structure, resulting in decreased extraction efficiency for polyphenols. As shown in Figure 4a, temperature also played a key role in determining the extraction efficiency of polyphenols. By increasing the extraction temperature from 25 °C to 60 °C, it was possible to identify an extraction profile that led to a progressively higher TPC.

The increase in temperature led to a decrease in the viscosity of the eutectic mixture, thereby reducing the limitations of mass transfer. Rebocho et al. [57] reported this phenomenon in their study on the fractionated extraction of polyphenols from mate tea leaves using a combination of hydrophobic/hydrophilic NADES. They found that this approach increased the total phenolic content at higher extraction temperatures. Additionally, the use of ultrasound systems, as reported in Lanjekar et al., further enhanced this effect [58]. Experimental findings of this work align with the existing literature, which generally suggests that increasing the operating temperature improves extraction efficiency. This is attributed to the higher solubility and diffusion coefficients of polyphenols in solvents, and the reduced surface tension and viscosity of the solvent mixture [50].

The assessment of the appropriate solvent-to-matrix ratio is a critical factor in this type of extraction, as an excess of solvent results in poorly concentrated extracts and excessive consumption of the extracting mixture. On the other hand, a solvent-to-matrix ratio that is too low can impede the diffusion of analytes and hinders the optimization of their extraction. This evidence is supported by Bubalo et al. and Ozturk et al., who used deep eutectic solvents for the green extraction of grape skin phenolics and the extraction of polyphenolic antioxidants from orange peel waste, respectively [50,59]. As depicted in Figure 4b, evaluating the extraction trend of polyphenols at different ratios reveals that the 1:10 ratio yields the highest content of polyphenols. However, increasing the quantity of solvent leads to a progressive decrease in TPC, a trend that is consistent with previous studies on hydroethanolic UAE.

**Figure 5:**
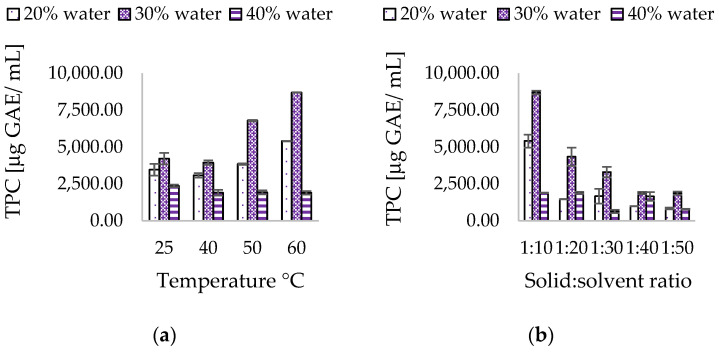
Effect of water content, (**a**) temperature, and (**b**) solid-to-solvent ratio in ChCl:LA NADES.

Results comparable to those achieved with ChCl:CA can be observed in experiments involving ChCl:LA, where maximum TPC is obtained under identical extraction conditions utilizing ChCl:CA. In fact, when the STF231 matrix was extracted with a ChCl:LA mixture, the highest yield of 8687.72 ± 127.44 μg GAE/mL was obtained by operating at 60 °C with a solvent-to-matrix ratio of 1:10 and 37 kHz sonication for 60 min, as depicted in Figure 5. Similarly, an increase in water content in the extraction mixture led to an increase in TPC, from 20% to 30% *w*/*v* of water, followed by a decrease when the water content reached 40% *w*/*v*. The addition of water resulted in a dilution effect, causing a slight decrease in viscosity, which was measured at 69.6 cPs at 20% water, 57.4 cPs at 30% water, and 53.8 cPs at 40% water. The rise in temperature also contributed to an increase in TPC, as observed in ChCl:LA-based NADES, with the maximum reached at 60 °C, as previously mentioned. The solvent-to-matrix ratio exhibited a similar trend as ChCl:CA at 30% *w*/*v* water at 60 °C, where the maximum extraction of polyphenols occurred at a ratio of 1:10, followed by a significant decrease with further additions of the extraction mixture. Among the various types of extracting agents studied for the extraction of polyphenols from STF231, it was evident that the combination of lactic acid and choline chloride in a ratio of 5:1 yielded the highest TPC. This finding is supported by several studies in the literature, where lactic acid-based NADES have proven to be superior in terms of extraction efficiency of polyphenols from aromatic plants, particularly when combined with ultrasound treatment [60,61].

**Figure 6:**
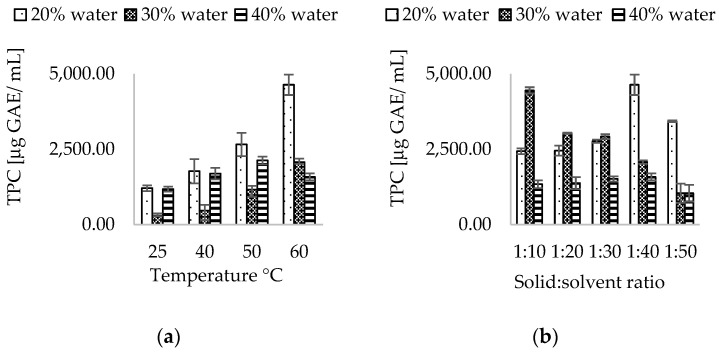
Effect of water content, (**a**) temperature, and (**b**) solid-to-solvent ratio in Su:CA NADES.

The extractions performed using Su:CA enabled comparable results to be highlighted with ChCl:CA, obtaining a TPC of 4638.83 ± 337.02 µg GAE/mL with 20% *w*/*v* water at 60 °C for 60 min of extraction at an ultrasound frequency of 37 kHz, but with a solid-to-solvent ratio of 1:40. As reported in Figure 6, the polyphenol extraction profile followed previous patterns, with the exception of the solid/solvent ratio and the amount of water, which in this case allowed for the highest quantity of polyphenols to be obtained with a 1:40 ratio and the use of 20% water.

**Figure 7:**
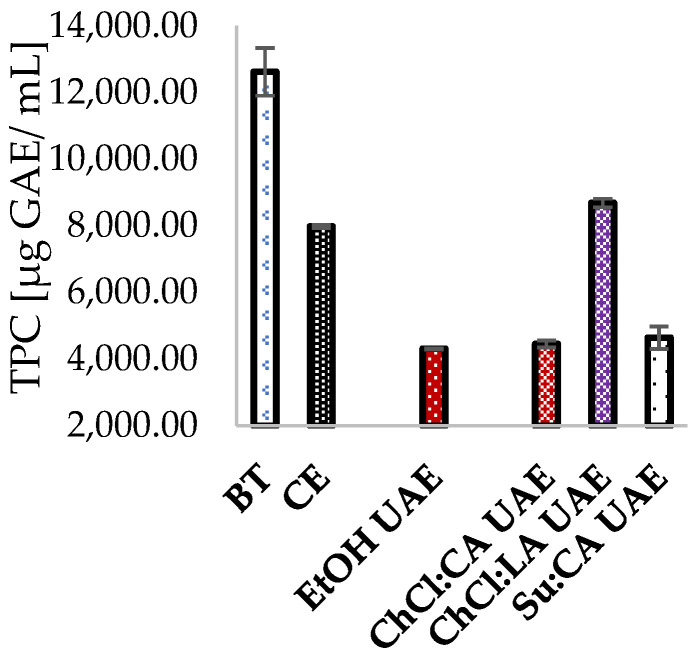
Comparison of TPC in original bitter tincture (BT) and TPC in best extraction conditions in conventional hydroethanolic extraction (CE), hydroethanolic ultrasound-assisted extraction (EtOH UAE), choline chloride/citric acid (ChCl:CA), choline chloride/lactic acid (ChCl:LA), and sucrose/citric acid (Su:CA).

Figure 7 presents a comparison of the TPC of the original bitter tincture with the polyphenol content obtained by using the extraction protocols studied and optimized under the parameters previously reported. As can be seen, hydroethanolic CE and ChCl:LA UAE yielded higher polyphenol content than other proposed methods, with 70.1% and 60.6% recovery of polyphenol content in the original bitter tincture, which contained 12,619.88 ± 715.05 µg GAE/mL. On the other hand, EtOH UAE, ChCl:CA, and Su:CA were less efficient in extracting polyphenols, as the TPC achieved was less than 40% of that of BT.

### 3.3. Antioxidant Activity

**Figure 8:**
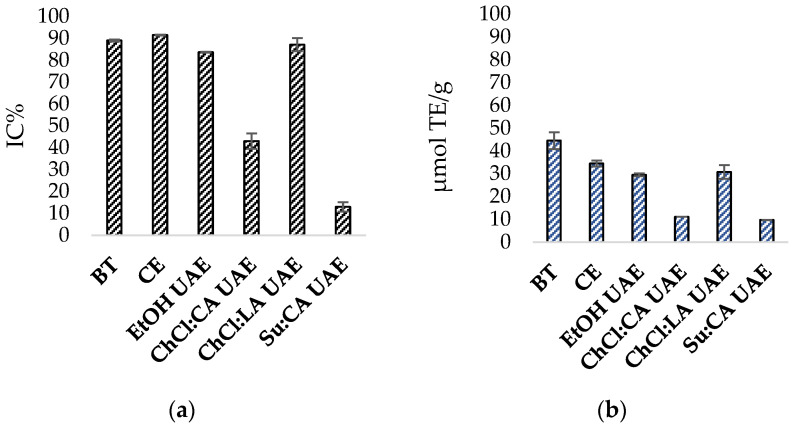
DPPH antioxidant activity (**a**) and FRAP reducing power (**b**) of original bitter tincture (BT), conventional hydroethanolic extraction (CE), hydroethanolic ultrasound-assisted extraction (EtOH UAE), choline chloride/citric acid ultrasound-assisted extraction (ChCl:CA UAE), choline chloride/lactic acid ultrasound-assisted extraction (ChCl:LA UAE), and sucrose/citric acid ultrasound-assisted extraction (Su:CA UAE).

From the comparative analysis of the antioxidant capacity in the best extraction conditions, it can be inferred that the conventional hydroethanolic extract had the highest ability to inhibit the DPPH radical, with IC% values of 91.45 ± 0.243, which was slightly higher than the 89.01 ± 0.42 of the original bitter tincture. Meanwhile, EtOH UAE and ChCl:LA UAE showed good antioxidant capacity, with 83.63 ± 0.16 IC% and 87.05 ± 2.98 IC%, respectively. On the other hand, low antioxidant capacity was observed in extracts obtained by ultrasound extraction with ChCl:CA and Su:CA mixtures, which reported IC% values of 42.99 ± 3.47% and 12.95 ± 2.12%, respectively, as illustrated in Figure 8a. The results obtained by the FRAP assay shown in Figure 8b aligned with those obtained by DPPH. Again, CE gave the highest result in terms of antioxidant capacity, with 34.48 ± 3.69 μmol TE/g. Hydroethanolic UAE and ChCl:LA UAE yielded comparable results of 29.52 ± 0.63 and 30.78 ± 3.04 μmol TE/g, respectively, while the other two eutectic mixtures exhibited the lowest antioxidant power values. The outcomes derived from the DPPH and FRAP tests demonstrated a strong correlation, as confirmed by the calculation of the Spearman coefficient R, which was 0.943 [62].

The necessity of performing DPPH and FRAP assays lies in the precise quantification of antioxidant activity, which, although predictable from the presence of polyphenols in the extracts, requires accurate determination.

### 3.4. HPLC Polyphenol Profiling

The original bitter tincture exhibited a high concentration of gallic acid, at 1.568 *±* 0.006 mg/mL, which made it the most abundant phenolic acid present. Additionally, the tincture contained significant amounts of rutin and trans-ferulic acid, in concentrations of 0.432 *±* 0.016 mg/mL and 0.266 *±* 0.005 mg/mL, respectively. When compared with other extracts, as shown in Table 3, the hydroethanolic CE method was able to recover a much higher quantity of quercetin, at 0.148 *±* 0.001 mg/mL, compared with the original tincture, which contained only 0.082 *±* 0.005 mg/mL. This aligned with the findings of the Folin–Ciocalteu test, which demonstrated the efficiency of the hydroethanolic CE method in extracting significant amounts of phenolic compounds from the STF231 matrix. Under the best extraction conditions operated in the EtOH UAE, all phenolic markers were identified, although at lower concentrations than in the CE. The titers of polyphenols in extractions mediated by choline chloride and lactic acid, under the previously mentioned conditions, were particularly promising. Comparing the ChCl:LA UAE extraction with the hydroethanolic UAE, no chlorogenic acid or rutin were found. However, all other phenolic markers, excluding gallic acid, were present in greater concentrations, as detailed in Table 3. It is worth noting that the caffeic acid content in ChCl:CA extracts was 0.254 ± 0.009 mg/mL, which exceeded that of all other extracts and was also higher than in the original bitter tincture and quercetin. Only rutin and ellagic acid were identified in ChCl:CA extracts, even if at relatively low concentrations compared with other extracts, but higher than in the hydroethanolic UAE. In Su:CA UAE extracts, only ellagic acid was detected and quantified at a concentration of 0.057 ± 0.001 mg/mL, which was higher than the hydroethanolic UAE and ChCl:CA UAE, but lower than the remaining extractions. The present findings on the analysis and quantification of phenolic markers aligned with the outcomes obtained from the Folin–Ciocalteu test, which demonstrated higher TPCs in hydroethanolic CE and ChCl:LA UAE under conditions mentioned previously. These conclusions corroborate previous studies in the literature where the use of NADES based on choline chloride and lactic acid have proven to be an effective method for extracting phenolic compounds, with efficiency comparable to methanol, which served as the control solvent [63,64,65].

### 3.5. HPLC Determination of Iridoids

The quantification of swertiamarin, gentiopicroside, sweroside, and amarogentin was determined for the extracts obtained under the optimized extraction conditions and in the original bitter tincture, as illustrated in Table 4. The highest swertiamarin content was identified in BT, amounting to 1.886 ± 0.013 mg/L, while significant concentrations of the analyte were also observed in the ChCl:LA UAE extract, which contained 1.626 ± 0.014 mg/L. The greatest quantity of gentiopicroside was surprisingly found in the ChCl:LA UAE extracts, extracted with a concentration of 2.506 ± 0.001 mg/L, while the CE allowed for more effective isolation of sweroside and amarogentin from the STF231 by-product. Su:CA extracts were found to be ineffective in recovering any of the target analytes, while ChCl:CA UAE extracts showed less effectiveness in extracting iridoids when compared with ChCl:LA UAE extracts. The possibility of employing eutectic mixtures based on choline chloride and lactic acid for the removal of iridoid glycosides proposed in this study aligned with other studies in the literature, where choline chloride, lactic acid, and water mixtures have been highlighted as particularly effective in the recovery of this class of molecules, with efficiencies comparable to conventional extractions based on hydromethanolic mixtures [65]. The utilization of NADES, which generates cavitation and interacts with cell walls to promote their disintegration, allows for the extraction of substantial amounts of iridoids from depleted matrices. This method employs environmentally friendly and potentially reusable solvents, which distinguishes it as a promising approach.

### 3.6. Green Assessment

A comprehensive assessment of the extraction methods employed in specific conditions for CE, EtOH UAE, and NADES UAE was undertaken utilizing the GAPI tool. This resource facilitates a visual analysis of the sustainability of each phase associated with the process through a series of five pentagrams divided into subsections. Each color in the pentagrams is representative of the impact of each individual operation on the overall process, with green indicating a low impact, red signifying a high impact, and yellow indicating a medium impact. The phases of collection, storage, transport, and conservation of the sample are represented in Sections 1–4, while Section 5 focuses on the investigation method, specifically whether it is direct or indirect and qualitative, quantitative, or both. Sections 6–8 assess the extraction procedures, the use of reagents, and sample pretreatments. Sections 9–11 concern the safety of the process in terms of chemical usage, the quantities utilized, and the potential risk to human health. Lastly, Sections 12–15 evaluate the equipment utilized, the energy consumed during operations, the occupational risks, waste generation, and their management [36].

**Figure 9:**
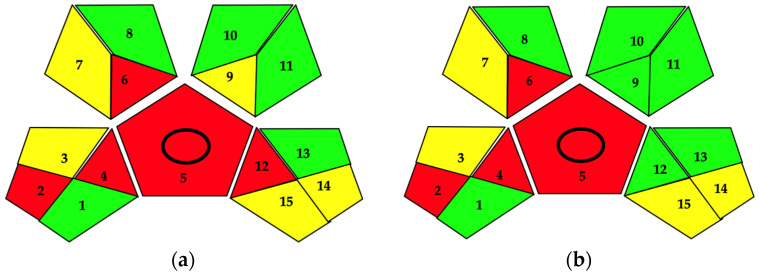
Green analytical procedure index evaluation of CE (**a**) and EtOH UAE, NADES UAE (**b**).

As depicted in Figure 9, each section boasts a green box, signifying a positive sustainability profile for the entire process. Although the green assessment for UAE extractions, both hydroethanolic and mediated by NADES, overlaps, the CE-related pictograms exhibit some disparities, particularly in Sections 9 and 12.

Section 9 regards the quantity of solvents and agents utilized, which, in the case of CE, surpasses 10 mL. This larger volume is necessary to process a sufficient amount of extract for subsequent analytical determinations. In contrast, UAE extractions can achieve the same results with samples under 10 mL. According to GAPI metrics, extractions using volumes less than 10 mL are preferred, marked in green for hydroethanolic UAE and NADES. Furthermore, Section 12 of the CE pictogram is marked in red, while it is green for UAE, reflecting the process’s energy consumption. CE necessitates a stirring and heating system to operate for 48 h to obtain an extract, whereas UAE only requires 1 h and can process multiple samples simultaneously, making it seem less sustainable when compared with UAE extractions.

**Figure 10:**
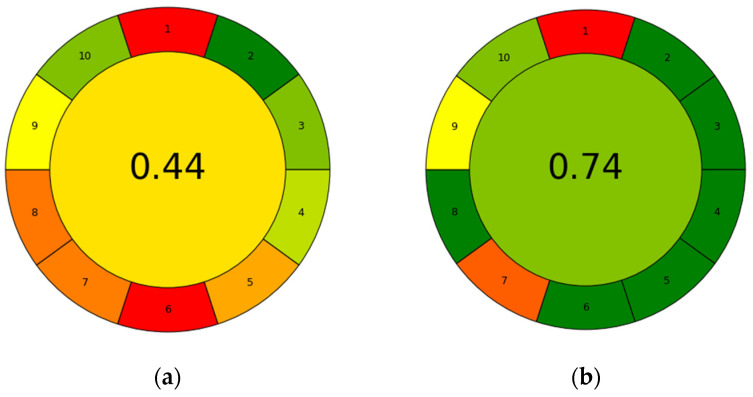
AGREEprep assessment of CE (**a**) and EtOH UAE, NADES UAE (**b**). Numbers refer to: 1—sample preparation placement, 2—hazardous material, 3—sustainability, renewability, and reusability materials, 4—waste, 5—size economy of the sample, 6—sample throughput, 7—integration and automation, 8—energy consumption, 9—post-sample configuration for the analysis, 10—operator’s safety.

The complementary assessment was conducted using AGREEprep, a tool that focuses on evaluating the sustainability profile of the sample preparation phase. While GAPI provides an overview that covers sampling to analytical measurements, AGREEprep offers a complementary and detailed green assessment specifically for the extraction methods used in this study. In fact, sample preparation appears to be the most critical step due to the use of solvents, reagents, strong acids and bases, and energy requirements, and it significantly impacts the global sustainability of the process. This metric evaluates 10 criteria by assigning scores between 0 and 1. The results are shown on a final graph, highlighting the impact of each evaluated phase, the overall impact, and the relative score using a colorimetric scale. Scores approaching 1 indicate methods that align well with green chemistry principles, whereas scores closer to 0 indicate methods that do not [37]. The comparative analysis conducted using the AGREEprep tool demonstrated that the natural eutectic solvent extraction method coupled with ultrasound was more environmentally friendly than hydroethanolic extraction, with a sample preparation score of 0.74 compared with 0.44 (Figure 10), as detailed in Table S1. This analysis underscores the significant impact of the sample preparation phase on the overall sustainability of the process. Therefore, it is crucial to develop effective tools for investigating critical issues. The AGREEprep tool identified several critical points in ultrasound-assisted extractions using NADES, including the ex situ sample preparation location, which required transport to the treatment and analysis site (point 1), the lack of automation in the sample preparation process (point 7), and the preference for alternative analytical investigation methods over HPLC in point 9.

To support the outcomes obtained, the AGREE tool was ultimately employed, which facilitated the assessment of environmental and occupational hazards related to the entire process and was grounded in the 12 principles of green analytical chemistry [38].

**Figure 11:**
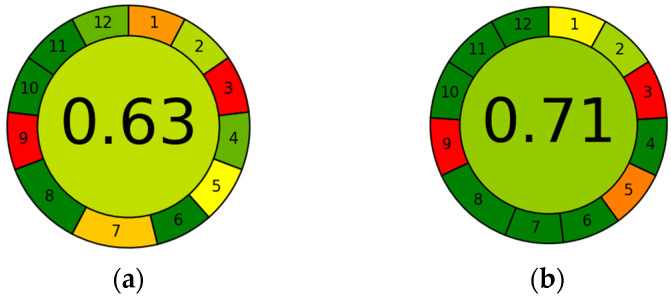
AGREE assessment of CE (**a**) and EtOH UAE, NADES UAE (**b**). Numbers refer to: 1—avoid sample treatment, 2—minimal sample size, 3—in situ measurements, 4—save reagents, 5—automated and miniaturized methods, 6—derivatization avoided, 7—waste avoided, 8—multianalyte methods, 9—energy minimized, 10—reagents from renewable source, 11—toxic reagents eliminated, 12—operator safety.

Hydroethanolic extraction and ultrasound-assisted extraction mediated by NADES demonstrated a high level of sustainability, with a score of 0.63 for CE and a score of 0.71 for UAE, as depicted in Figure 11. This finding was also supported by the study of Mir-Cerdà A et al. [66], in which hydroethanolic and NADES-based extractions were carried out, followed by the assessment of the sustainability profile using AGREE, with scores that were in close agreement with those obtained from the evaluation of the extraction processes in this study. The AGREE assessment showed no major differences on the sustainability profile for both CE and UAE. This outcome was expected because AGREE evaluated the entire process as a whole and did not specifically focus on the extraction method used.

Considering the evaluations and assessments carried out, it can be concluded that the extractions carried out in this study exhibit a considerable level of ecological stability, particularly in the UAE, where the difference of scores obtained with AGREEprep is marked between CE and UAE, especially if compared with the assessment performed with the AGREE framework. This is achieved by employing lesser amounts of solvents in UAE, predominantly renewable or recyclable, minimizing waste, and simultaneously processing numerous samples. The preparatory phase of these samples reduces energy waste and occupational hazards. Further advancements can be made to enhance the sustainability of the proposed processes. This can be accomplished by incorporating predictive tools that decrease the number of experimental tests required and by scaling down extraction procedures to further decrease waste. Although additional improvements are necessary, the overall direction appears promising. The integration of NADES and the UAE methods allows to produce extracts containing significant concentrations of the target analytes while minimizing environmental impact.

## 4. Conclusions

This study presents an innovative approach for the upcycling of STF231, a by-product of the medicinal extracts industry. By focusing on the recovery of polyphenols and iridoids from an already exhausted matrix, the research has adopted a comparative approach in evaluating conventional and modern extraction techniques. Specifically, the study has shown promising results when using natural deep eutectic solvents, such as a mixture of choline chloride and lactic acid, in combination with ultrasound-assisted extraction. The optimal conditions for this method were found to be a 5:1 ratio of choline chloride and lactic acid, with the addition of 30% water, and a matrix/solvent ratio of 1:10, extracted at 60 °C for 60 min. Under these conditions, the extracts obtained had a total polyphenol content of 8687.72 ± 127.44 µg GAE/mL and reasonable antioxidant activity comparable to conventional hydroethanolic extraction. This study also identifies significant concentrations of caffeic acid and gentiopicroside in the extracts, making them suitable for use in health preparations and demonstrating a high level of sustainability.

## Figures and Tables

**Table 1:** Composition of NADES.

Component 1	Component 2	NADES	Ratio	% Water ^a^
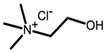 choline chloride	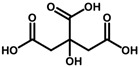 citric acid	ChCl:CA	1:2	20 30 40
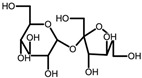 sucrose	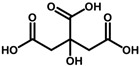 citric acid	Su:CA	1:2	20 30 40
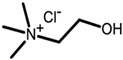 choline chloride	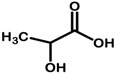 lactic acid	ChCl:LA	1:5	20 30 40

^a^ expressed as *w*/*v.*

**Table 2:** Composition of STF 231.

STF 231	Content ^a^	Unit
Moisture (fresh product)	48.08 ± 0.01	% dm
Moisture (dried product)	8.72 ± 0.00	% dm
Ash	5.64 ± 0.54	% dm
Total nitrogen	0.03 ± 0.00	% dm
Total proteins	2.29 ± 0.61	% dm
Crude fats	6.96 ± 0.83	% dm
Total cellulose	25.88 ± 1.33	% dm
Glucose	0.33 ± 0.00	gL^−1^
Fructose	0.68 ± 0.01	gL^−1^
Sorbitol	0.078 ± 0.00	gL^−1^

^a^ expressed as the average of triplicates ± SD.

**Table 3:** Polyphenol composition of original bitter tincture (BT), conventional hydroethanolic extraction (CE), hydroethanolic ultrasound-assisted extraction (EtOH UAE), choline chloride/citric acid ultrasound-assisted extraction (ChCl:CA UAE), choline chloride/lactic acid ultrasound-assisted extraction (ChCl:LA UAE), and sucrose/citric acid ultrasound-assisted extraction (Su:CA UAE).

Phenolic Compound	Linear Equation	R^2^	t_R_	LOD	LOQ			Concentration			
						BT ^1^	CE ^1^	EtOH UAE ^1^	ChCl:CA UAE ^1^	ChCl:LA UAE ^1^	Su:CA UAE ^1^
Gallic acid	y = 3017.8x + 67.465	0.999	3.980	0.020	0.063	1.568 ± 0.006	0.644 ± 0.003	0.236 ± 0.005	n.d.	0.137 ± 0.003	n.d.
Chlorogenic acid	y = 11,712x − 14.077	0.992	7.408	0.002	0.007	0.047 ± 0.000	0.061 ± 0.000	0.019 ± 0.001	n.d.	n.d.	n.d.
Caffeic acid	y = 9084.1x + 7.5368	0.994	9.153	0.006	0.020	0.171 ± 0.003	0.115 ± 0.002	0.045 ± 0.000	n.d.	0.254 ± 0.009	n.d.
Rutin	y = 7127x − 0.9877	0.999	11.793	0.002	0.007	0.432 ± 0.016	0.364 ± 0.000	0.019 ± 0.000	0.022 ± 0.000	n.d.	n.d.
Ellagic acid	y = 26,294x + 54.986	0.999	13.736	0.002	0.007	0.151 ± 0.001	0.106 ± 0.001	0.013 ± 0.001	0.018 ± 0.000	0.075 ± 0.000	0.057 ± 0.001
Trans-ferulic acid	y = 13,763x − 199.26	0.995	14.630	0.017	0.053	0.266 ± 0.005	0.184 ± 0.006	0.067 ± 0.002	n.d.	0.112 ± 0.000	n.d.
Quercetin	y = 13,837x + 11.29	0.999	17.407	0.009	0.027	0.082 ± 0005	0.148 ± 0.001	0.030 ± 0.000	n.d.	0.094 ± 0.002	n.d.

^1^ result expressed in mg/mL as the average of at least a triple injection of the sample ± SD.

**Table 4:** Iridoids profiling in original bitter tincture (BT), conventional hydroethanolic extraction (CE), hydroethanolic ultrasound-assisted extraction (EtOH UAE), choline chloride/citric acid ultrasound-assisted extraction (ChCl:CA UAE), choline chloride/lactic acid ultrasound-assisted extraction (ChCl:LA UAE), and sucrose/citric acid ultrasound assisted extraction (Su:CA UAE).

Iridoids	Linear Equation	R^2^	t_R_	LOD	LOQ			Concentration			
						BT ^1^	CE ^1^	EtOH UAE ^1^	ChCl:CA ^1^ UAE	ChCl:LA ^1^ UAE	Su:CA ^1^ UAE
Swertiamarin	y = 7 × 10^6^x + 1071.2	0.999	10.653	0.008	0.025	1.886 ± 0.013	0.0366 ± 0.002	0.406 ± 0.018	0.112 ± 0.014	1.626 ± 0.020	n.d.
Gentiopicroside	y = 3 × 10^6^x + 20,893	0.999	11.523	0.012	0.037	1.076 ± 0.016	0.237 ± 0.000	0.288 ± 0.020	0.076 ± 0.005	2.506 ± 0.001	n.d.
Sweroside	y = 3 × 10^6^x + 5645.2	0.999	12.006	0.009	0.029	1.211 ± 0.005	3.119 ± 0.056	0.968 ± 0.108	0.274 ± 0.028	1.664 ± 0.074	n.d.
Amarogentin	y = 1 × 10^7^x − 16,003	0.999	19.6967	0.010	0.032	0.433 ± 0.005	0.651 ± 0.005	0.340 ± 0.083	0.182 ± 0.064	0.304 ± 0.001	n.d.

^1^ result expressed in mg/L as the average of at least a triple injection of the sample ± SD.

## Data Availability

The original contributions presented in the study are included in the article/Supplementary Material; further inquiries can be directed to the corresponding author.

## Supplementary Materials

The following supporting information can be downloaded at: https://www.mdpi.com/article/10.3390/antiox13081014/s1, Figure S1: FTIR of citric acid, choline chloride, and ChCl:CA; Figure S2: FTIR of citric acid, sucrose, and Su:CA; Figure S3: FTIR of lactic acid, choline chloride, and ChCl:LA; Table S1: AGREEprep assessment score detail.
