# Supplementary material for: A Sustainable Approach to Valuable Polyphenol and Iridoid Antioxidants from Medicinal Plant By-Products
Source: Antioxidants (Basel). 2024 Aug 20;13(8):1014. doi: 10.3390/antiox13081014 (PMC11351505; doi:10.3390/antiox13081014)
Supplement: Supplementary file 1 [file antioxidants-13-01014-s001.zip › antioxidants-3145350-supplementary.pdf]

Supplementary materials

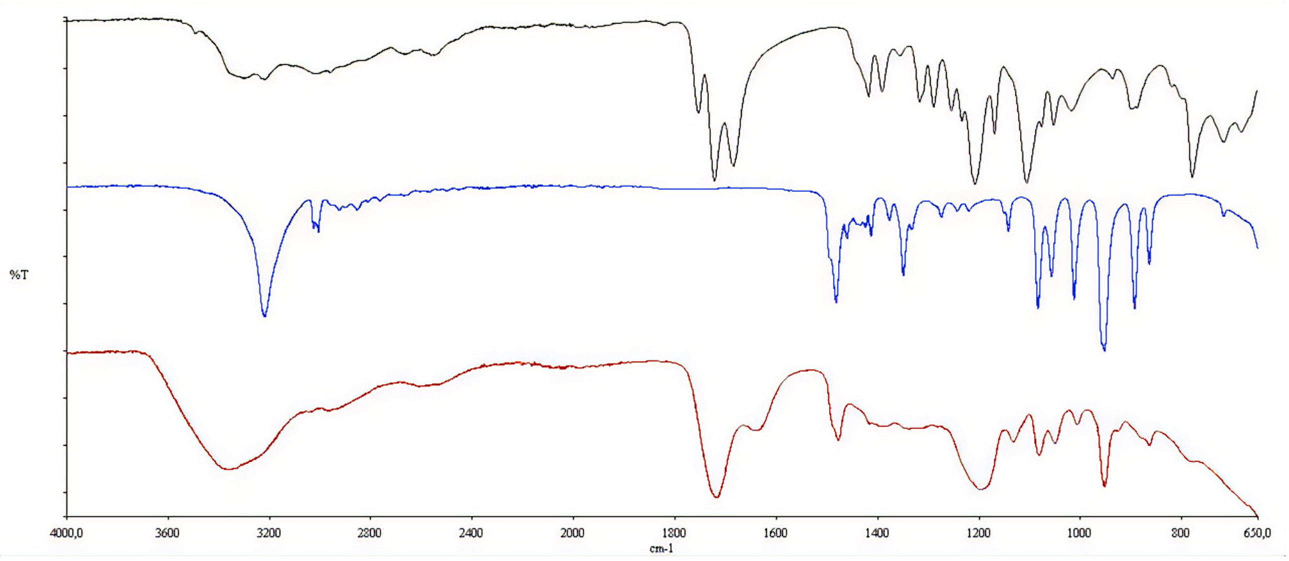

Figure S1: FTIR of citric acid (black line) choline chloride (blue line) and ChCl:CA (red line)

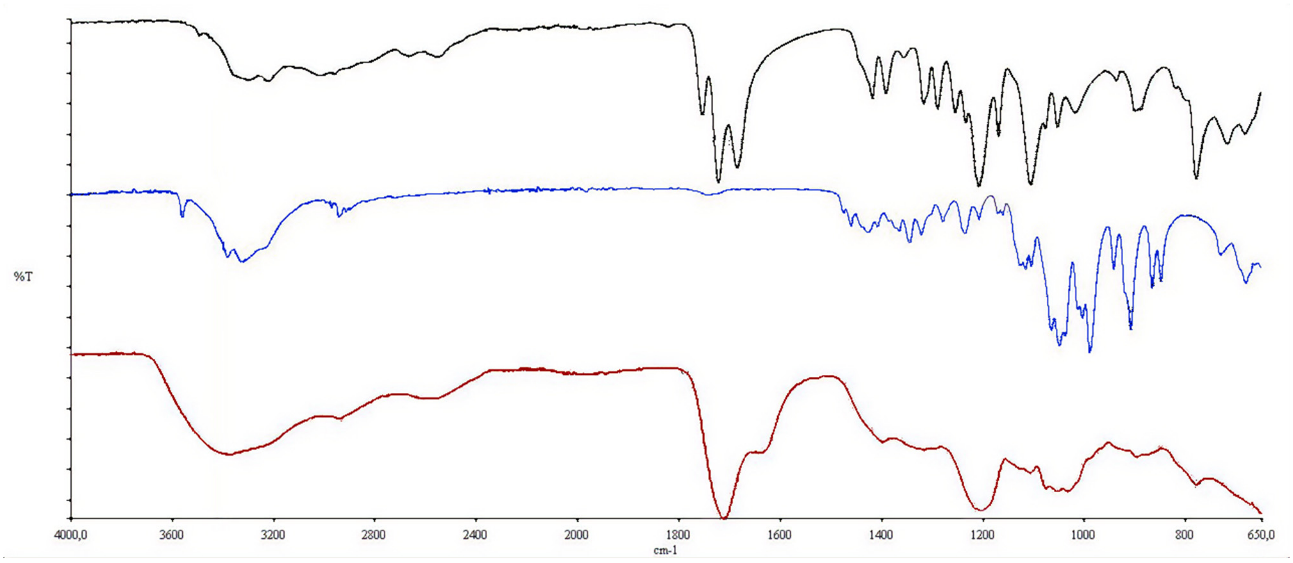

Figure S2: FTIR of citric acid (black line) sucrose (blue line) and Su:CA (red line)

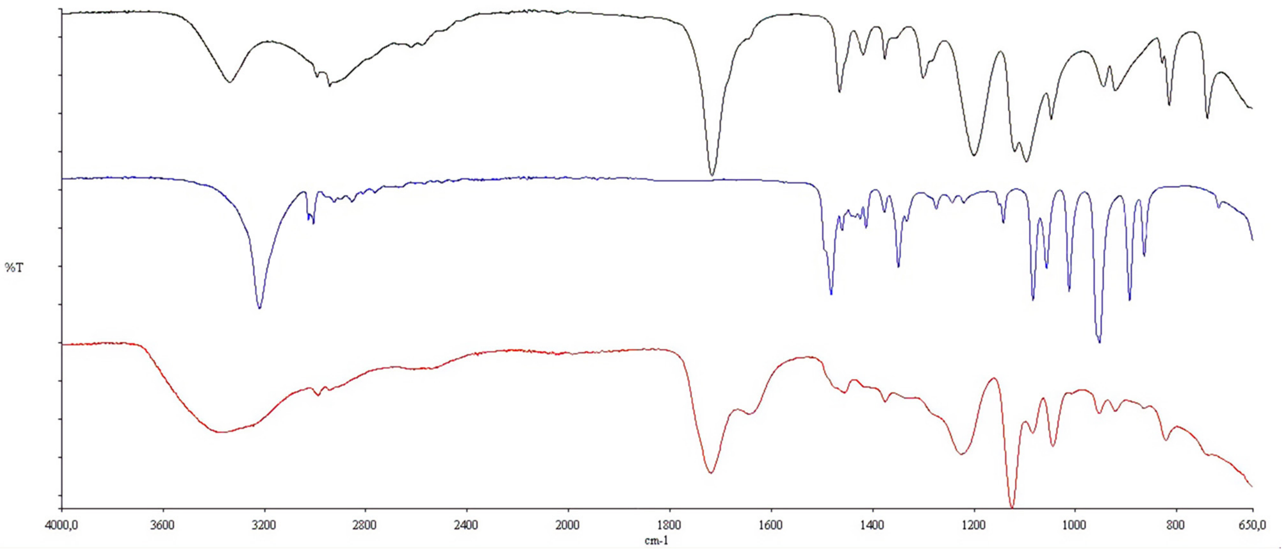

Figure S3: FTIR of lactic acid (black line) choline chloride (blue line) and ChCl:LA (red line)

Table S4: AGREEprep assessment report detail for CE and UAE

Criterion	CE score	UAE score	Weight	Equation	Reference
**1. Sample preparation placement**	0.00	0,00	1	-	[37]
**2. Hazardous materials**	0.94	1,00	1	Score = -0,145 x ln(amount of hazardous substances in g or mL) + 0,3333 [37]	[37]
**3. Sustainability, renewability, and reusability of materials**	0.75	1,00	1	-	[37]
**4. Waste**	0.67	1,00	1	Score = -0,161 x ln(sample mass or volume in g or mL) + 0,6295	[37]
**5. Size economy of the sample**	0.23	1,00	1	Score = -0,145 x ln(sample mass or volume in g or mL) + 0,6667	[37]
**6. Sample throughput**	0.00	0,98	1	Score = 0,2354 x ln(number of prepared samples per hour)	[37]
**7. Integration and automation**	0.25	0.25	1	-	[37]
**8. Energy consumption**	0.31	0.90	1	-	[37]
**9. Post-sample preparation configuration for analysis**	0.50	0,50	1	-	[37]
**10. Operator's safety**	0.75	0,75	1	-	[37]
